# A 63 element 1.75 dimensional ultrasound phased array for the treatment of benign prostatic hyperplasia

**DOI:** 10.1186/1475-925X-4-39

**Published:** 2005-06-17

**Authors:** Khaldon Y Saleh, Nadine Barrie Smith

**Affiliations:** 1 Department of Bioengineering Graduate Program in Acoustics College of EngineeringThe Pennsylvania State University 206 Hallowell Building University Park, PA 16802, USA; 2 Graduate Program in Acoustics College of Engineering The Pennsylvania State University 206 Hallowell Building University Park, PA 16802, USA

**Keywords:** ultrasound transducer, 1.75 dimensional array, focusing, matching layer

## Abstract

**Background:**

Prostate cancer and benign prostatic hyperplasia are very common diseases in older American men, thus having a reliable treatment modality for both diseases is of great importance. The currently used treating options, mainly surgical ones, have numerous complications, which include the many side effects that accompany such procedures, besides the invasive nature of such techniques. Focused ultrasound is a relatively new treating modality that is showing promising results in treating prostate cancer and benign prostatic hyperplasia. Thus this technique is gaining more attention in the past decade as a non-invasive method to treat both diseases.

**Methods:**

In this paper, the design, construction and evaluation of a 1.75 dimensional ultrasound phased array to be used for treating prostate cancer and benign prostatic hyperplasia is presented. With this array, the position of the focus can be controlled by changing the electrical power and phase to the individual elements for electronically focusing and steering in a three dimensional volume. The array was designed with a maximum steering angle of ± 13.5° in the transverse direction and a maximum depth of penetration of 11 cm, which allows the treatment of large prostates. The transducer piezoelectric ceramic, matching layers and cable impedance have been designed for maximum power transfer to tissue.

**Results:**

To verify the capability of the transducer for focusing and steering, exposimetry was performed and the results correlated well with the calculated field. *Ex vivo *experiments using bovine tissue were performed with various lesion sizes and indicated the capability of the transducer to ablate tissue using short sonications.

**Conclusion:**

A 1.75 dimensional array, that overcame the drawbacks associated with one-dimensional arrays, has been designed, built and successfully tested. Design issues, such as cable and ceramic capacitances, were taken into account when designing this array. The final prototype overcame also the problem of generating grating lobes at unwanted locations by tapering the array elements.

## 1. Background

Treating prostate diseases such as prostate cancer and benign prostatic hyperplasia (BPH) is of great importance. In the United States, most of the new diagnosed prostate cancer cases appear in men who are over the age of 55 while most of the BPH cases appear after the age of 60. According to the National Cancer Institute, 50 percent of men between the ages of 60 and 70, and 90 percent of men between the ages of 70 and 90, have BPH symptoms. Prostate cancer is a life threatening disease while BPH is a benign growth that needs to be treated since normal urine flow can be blocked as a result of the prostate pushing against the urethra and the bladder (National Cancer Institute 1999).

Existing techniques for treating such diseases include hyperthermia, focus surgery, radiotherapy, chemotherapy and surgery. Currently, surgical techniques are widely used over the other modalities; that is due to the inefficiency and the unpleasant side effects those modalities have. However surgical techniques have numerous complications that appear in about one in four cases, which include impotence, incontinence, and urinary tract infections and often require lengthy hospitalization [1,2].

Due to its noninvasiveness, focus surgery is gaining more attention than the other modalities in the past decade [3]. With focus surgery, ultrasound or microwave devices are used to generate a focused beam at a certain location in the prostate, which kills the cells at that location by raising their temperature to 60°C for about ten seconds. Attention is given more to ultrasound rather than microwave. That is because microwave has either a shallow penetration depth (when high frequencies are used) or a lack of the ability to generate a significant focus (when low frequencies are used) [4].

With focused ultrasound (FUS), tissue is noninvasively ablated by elevating the temperature at the focal point above 60°C using short sonications (10–30 seconds). In this kind of treatment, the target volume can be ablated by focusing the ultrasound beam at a certain position, and then steering the focus to cover the whole enlarged volume. Thus FUS can be used for prostate ablation to remove a non-desirable growth of the prostate [5-7]. Since the tissue volume to be ablated is larger than the geometric focus of the array, the transducer needs to be moved repeatedly to destroy the desired volume and unnecessarily extend the treatment time. Phased arrays overcome this problem by electrically steering the focal point from one location to another by changing the phase and power to the individual elements of the array. Previous effective prostate ultrasound devices include both mechanically and electrically steered designs. Electrically steered include a one-dimensional (1-D) 120 × 1 aperiodic, linear array design (90 × 15 mm^2^) which reduced grating lobes and could steer the focus in the radial and transverse but not the longitudinal direction [8]. Another experimental design was a 62 × 1, linear array (75 × 15 mm^2^) with a mechanical translation that could electrically steer the focus in the radial and transverse but not the longitudinal direction [9]. The drawbacks behind these designs are that they can only steer the focus in the radial and transverse directions or require complex mechanisms to move the focus. Improvements over 1-D arrays for the treatment of localized prostatic cancer can be achieved. Many multi-dimensional ultrasound phased arrays have been designed and built for the treatment of prostate diseases; that includes a 1.5-dimensional (1.5-D) phased array [10] (a 1.5-D array consists of three individual linear array that can be driven individually or connected together to form a single linear array), a 1.75-dimensional (1.75-D) phased array [11] (a 1.75-D array consists of many individual linear arrays that are driven separately), and a two-dimensional (2-D) phased array [12]. The advantage with a multi-dimensional phased array is that it has the capability of focusing and steering in a 3-dimensional (3-D) representation of the prostate without the need to physically move the array.

Issues regarding the construction of an array used for FUS of the prostate initially deal with the frequency and size of the ceramic to be diced into an array. The resonant frequency should be greater than 500 kHz [13] while the size of the transducer needs to be large enough to be able to deliver high power but small enough to be an intracavitary device. Before construction, computer simulations can be performed to determine the acoustic field. Pressure wave and temperature simulations indicated that a tapered array design reduced grating lobes significantly compared to equal element size arrays. Based on the computer model, a tapered array that satisfied grating lobes, frequency, and size limitations was designed. Lead zirconate titanate (PZT- 8) was chosen as the ceramic material of the array since it has the capability of handling the high electrical powers used in focused ultrasound. To maximize the acoustical power transmission from the elements and improve the structural integrity of the array face, two matching layers were designed and fabricated. Issues regarding the cabling and electrical matching of the elements were also considered. Exposimetry of the acoustic field from the array was performed to compare experimental and calculated theoretical results. *Ex vivo *experiments using bovine tissue were also performed to demonstrate the feasibility of the array to ablate tissue. This paper describes the design, construction and evaluation of a 1.75-D ultrasound phased array that is capable of focusing and steering in a 3-D volume to be used in the treatment of BPH.

## 2. Methods

### 2.1 Simulations

#### 2.1.1 Acoustic pressure field simulations

**Figure 1:**
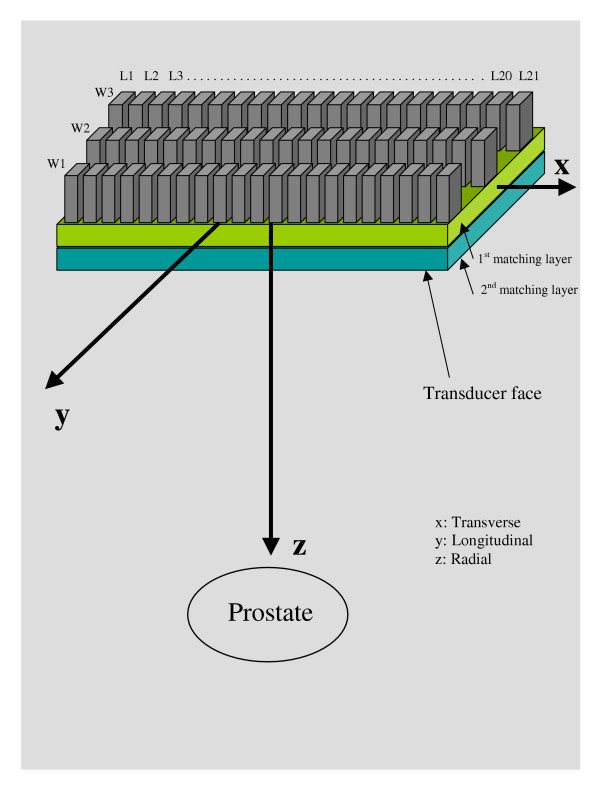
Based on the simulations, a diagram of the 1.75-D 63 element (3 × 21) tapered array with total size of 27 × 53 mm^2 ^with the proportions of the ceramic and matching layer illustrated. The diced face of the ceramic was cut 100% through and each individual element was attached to the electrical cabling using low temperature soldering material.



MATLAB computer simulation programs were written to determine the number and the size of the phased array elements in addition to determining the pressure and temperature fields from the device. The array was modeled (Figure 1) as a 1.75-D tapered array in order to have focusing and steering capabilities in both x and z directions (x = transverse, y = longitudinal and z = radial). Focusing in the y direction is done in a different way; the array is divided into three identical rows, each one represents a single linear array. If the focus is required at y = 0, the middle row should be used. A focus at y = -0.9 cm requires driving the lower row, while a focus at y = +0.9 cm requires the operation of the upper row. Although the degree of freedom in the y direction is not perfect, the size of the lesion generated by a single sonication compensates for that, since the focus length is about 9 mm in the y direction. With these requirements, this array was capable of focusing and steering with a steering angle of ± 13.5° with maximum focal depth of 11 cm. The phase of each element was determined such that signals from individual elements were coherent at the focal point. Measuring the difference in path length between each element to the focus in comparison to the path from the center of the array to the focus determined the element phase calculation. The phase, φ_*i*_, (degrees) of element *i *was given by:

Where λ is the wavelength (m), *d*_*i *_is the distance (m) from the centre of element *i *to the focal point, *d*_*o*_is the distance (m) from the centre of the array to the focus and *n *is an integer to keep 0 ≤ φ_*i *_≥ 360°. Huygen's principle was used to model the pressure field as a summation of simple sources [14] and the total acoustic pressure at any point in the field was calculated using the discrete approximation of the Rayleigh- Sommerfeld equation:



Where *p *is the total acoustic pressure in Pascals (Pa), *P *is the total acoustic power emitted by the array in watts (W), ρ is the density of the medium (998 kg·m^-3^), *c *is the speed of sound (m·s^-1^), *A *is the total surface area of the array (m^2^), *f *is the resonant frequency (1.2 MHz), *S *is the area of the corresponding element (m^2^) and α is the attenuation in soft tissue (10 Np·m^-1^·MHz^-1^).

**Figure 2:**
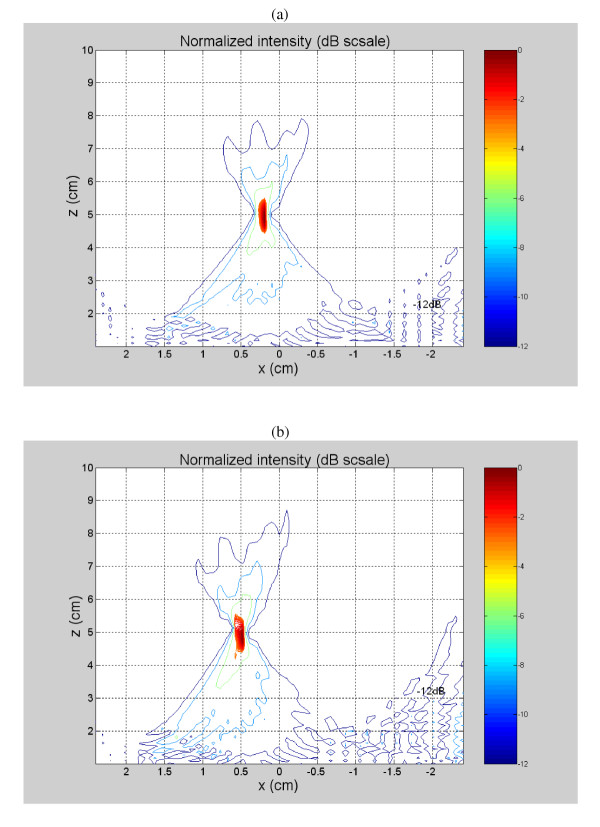
Off-axis focusing has a direct impact on the grating lobe level. Increasing the steering angle by changing the focal point position in the x direction increases the grating lobe level. For a focus aimed at (0.2, 0, 5) and (0.5, 0, 5) cm, a fair grating lobe level of about -12dB was observed, as seen in (a) and (b), respectively.

The acoustic pressure field simulations started with a 1-D model that was used to simulate different tapering techniques to see their effect on the grating lobe values. Equal, linear, Hanning and Hamming tapering techniques were simulated. Improvements to the tapered array design started with a 27 × 53 mm^2 ^solid piezoceramic cut into a 3 × 21 pattern with 63 individual elements with lengths (*L*_*i*_) of 1.68, 1.73, 1.81, 1.91, 2.02, 2.14, 2.26, 2.36, 2.43, 2.48, 2.50, 2.48, 2.43, 2.36, 2.26, 2.14, 2.02, 1.91, 1.81, 1.73, 1.68 mm for elements *i *= 1 through 21, respectively, and widths (*W*_*i*_) of 9.0 mm for all elements *i *= 1 through 3, respectively (Figure 1). The maximum possible steering angle was calculated to be tan^-1^(1.2/5.0) = 13.5° with maximal focal depth of 11 cm. Off-axis focusing and the grating lobe level are directly related to each other since increasing the steering angle causes a nonlinear increase in the grating lobe level. However, the designed array described in this paper kept a good grating lobe level when aiming the focus at a point that was 5 mm away from the z direction. When focusing at (0.2, 0, 5) and (0.5, 0, 5) cm, the grating lobe level was kept around -12 dB, as can be seen in Figures 2(a) and 2(b), respectively.

#### 2.1.2 Temperature distribution simulations

From the pressure field of the simulated array, the temperature distribution in the tissue was modeled using the Pennes' bioheat transfer equation (BHTE) [15]:



Where *C*_*t *_is the specific heat of the tissue (3770 J·kg^-1^·°C^-1^), *K *is the thermal conductivity (0.5 W·m^-1^·°C^-1^), *T *is the temperature at time *t *at the point x, y, z in °C, *T*_*a *_is the arterial blood temperature (37°C), *w *is the perfusion in the tissue in kg·m^-3^·s^-1^, *C*_*b *_is the specific heat of the blood (3770 J·kg^-1^·°C^-1^) and *q*(x, y, z) is the power deposited at the point x, y, z. The power was calculated from the pressure field of the array design while the BHTE was determined using a numerical finite difference method with the boundary conditions set at 37°C. The total intensity at point (x, y, z) was also calculated from the pressure field of the simulated array and is given by [16]:



Where *I*(x, y, z) is the intensity at point (x, y, z) in W·m^-2^.

**Figure 3:**
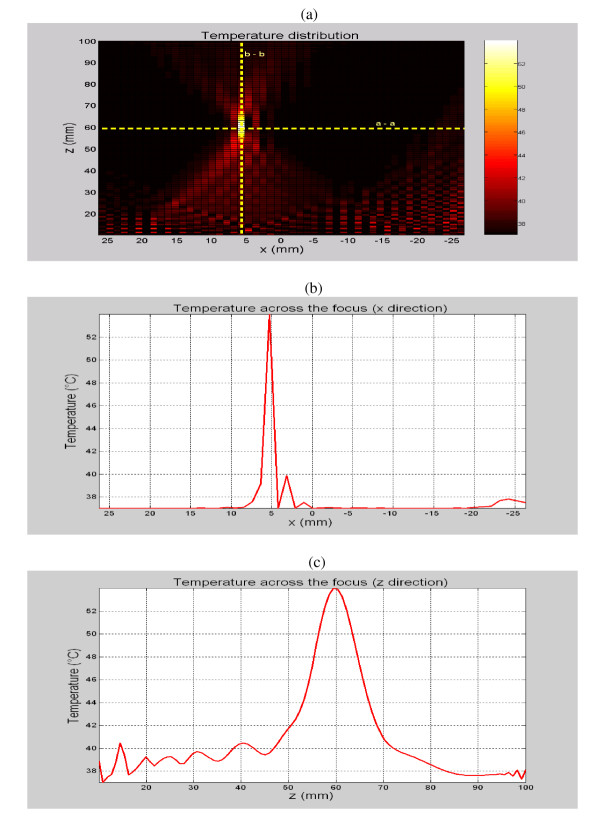
A temperature map (a) for a focus aimed at (5, 0, 60) mm and cross section temperatures (b) and (c) across the lines a-a and b-b, respectively, as a function of distances x and z, respectively.

Temperature simulations were used to verify the potential to increase the tissue temperature to about 60°C with short sonications. Both on- and off-axis simulations were performed to see what impact they have on grating lobe values. The effect of off-axis focusing on the temperature distributions becomes more evident at high steering angles. For the case where the steering angle was set to 4.75°, i.e., focus at (5, 0, 60) mm, the temperature distribution was calculated and plotted in Figures 3(a–c) as a distribution at the plane of interest, a cross section along the line a-a and a cross section along the line b-b, respectively. Those three figures show that the simulated temperature at the focal point was about 54°C, while the temperature elsewhere was kept below 41°C.

### 2.2 Transducer construction

The 1.75-D array described in this chapter vibrates in the thickness mode, which means that ε_33 _is the permittivity value of interest. Although Lead zirconate-titanate (PZT-5H) has a higher permittivity, which would lead to a higher capacitance, it cannot handle the large power that is used in FUS. PZT-4 and PZT-8 are good candidates concerning power, with an advantage for PZT-8 over PZT-4. The capacitance of a certain element in the array depends on the thickness (which is constant for all elements), the permittivity (which is a material characteristic) and the surface area of that element. Since the areas of the elements of the array are small, this will result in a small capacitance and thus large element impedance.

PZT-8 can handle the large electrical power needed for tissue ablation, has an extremely high mechanical quality factor and extremely low loss factor. Thus PZT-8 material (TRS Ceramics, State College, PA, USA) was chosen at a frequency of 1.2 MHz and diced, in house, into 3 × 21 elements forming the complete array. The cuts were made by dicing the material 100% through its thickness with a kerf width of 300 μm using a dicing saw (Model 780, K & S-Kulick and Soffa Industries, Willow Grove, PA, USA) in our lab. For maximum acoustical power transfer from the individual elements to the tissue, two matching layers were designed and constructed. The thickness and material selection of the matching layers were designed based on the solution to a four-layer problem (transducer, first matching layer, second matching layer, and tissue), which ensured the required maximum power transfer. The acoustic impedance of the two matching layers (*Z*_1 _and *Z*_2_) was calculated using a criterion determined by Fraser:

*Z*_1 _= (*Z*_*piezo*_)^4/7 ^(*Z*_*tissue*_)^3/7 ^    (5)

*Z*_2 _= (*Z*_*piezo*_)^1/7 ^(*Z*_*tissue*_)^6/7 ^    (6)

**Figure 4:**
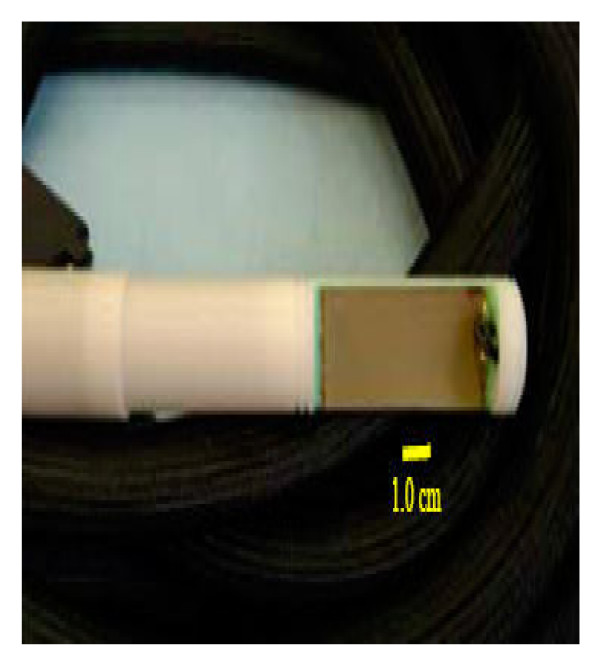
Photograph of the constructed, waterproof array with 7.0 m low capacitance cable that connected to the amplifier system.

Each of the two matching layers was designed for a quarter wavelength thickness. Accordingly, the thickness of the first and second matching layers was determined to be 0.396 and 0.429 mm, respectively. The first matching layer, mixed in-housed, was a 2:1, epoxy to silver mixture of Insulcast 501 (Insulcast, Roseland, NJ, USA) and 2–3 micron silver epoxy (Aldrich, Milwaukee, WI, USA), while the second matching layer was a SPURR (Spi Supplies, West Chester, PA, USA) four-part low viscosity material. For this array design (Figure 4), the specially machined, waterproof cylindrical applicator housing (30 mm diameter) was made from magnet compatible Delrin^® ^(Dupont, Wilmington, DE, USA) at the Penn State engineering shop.

Transmission line theory applies to the coaxial cables used in ultrasound applications. The simplest approximation for the transmission line is a lumped capacitance, where the total cable capacitance can be measured by multiplying the cable capacitance per meter, *Cm*, by the length of the cable *L *in meters. Since the load impedance (element impedance) is high, the cable has to be a low capacitance cable, which effectively means high cable impedance. A cable with a characteristic impedance of 75 Ω was found to be suitable

The 1.75-D array contains 63 elements. Since those elements are of different size, each one of them will have different electrical impedance depending on the surface area of that element which determines the capacitance of that element and thus the electrical impedance. The target is to match each one of these impedances to the common value of 50Ω∠0°. A simple *LC *(*L *= inductor, *C *= capacitor) impedance matching circuit was built for each of the 63 elements.

### 2.3 Exposimetry

To determine the acoustic field generated by the array, an automated computer controlled positioning system, which could translate a hydrophone (needle one with 0.5 mm diameter, Precision Acoustics, UK) throughout the acoustic field of the array placed in a water tank, was used. The transducer was submerged in water (room temperature, approximately 20°C) in a tank (120 × 50 × 52 cm^3^) made almost anechoic with sound absorbing rubber. A custom made degasser, built in-house, was used to reduce the dissolved oxygen content of the distilled water to 1–2 ppm to reduce cavitation. The system was controlled using a personal computer connected to a four-motor positioning system (Velmex Inc., Bloomfield, NY, USA) via the RS232 serial port and also connected, via the general purpose interface bus (GPIB), to a digital oscilloscope (Agilent 54622A, Agilent Technologies, Palo Alto, CA, USA) which recorded the voltage amplitudes detected by the hydrophone. Custom written, Quick Basic (Microsoft Corporation, Redmond, WA, USA) programs were used for automated control of the motors and data acquisition from the oscilloscope. Initially, multiple on-axis (i.e. where the focus is along the major z axis, z*f*) exposimetry experiments were performed. With the focus set to 0, 0, z*f *mm, z*f *was varied from 10 mm to 110 mm with a step size of 5 mm. To determine the repeatability or standard deviation of the focusing, 5–10 experiments were performed at each location. For off-axis studies (i.e., where the focus was not on z but aimed toward the x axis, x*f*), the focus was located at (x*f*, 0, 60) mm while the steering angle was adjusted to the desired value by choosing appropriate values for x*f*. The steering angle was varied from -12° to +12° with a step size of 2° in both x and y directions with multiple experiments (5–10) performed at each angle. In both the on-axis and off-axis experiments, the scanning step size was 0.5 mm while the scanning area was 40 × 40 mm^2^. The hydrophone voltage recordings were used to calculate the normalized intensities based on the pressures that were plotted as the mean and standard deviation of the results (x ± s.d.) and compared against the calculated values.

### 2.4 Ex vivo experiments

To test the ability of the array to generate lesions in non-perfused bovine tissue, the array was submerged 6 cm in water and aimed perpendicular to the surface of water. Fresh bovine tissue was obtained, placed in the water tank and held 2.5 cm in front of the array. For both on- and off-axis focusing, a single linear array was driven with an average electrical power of 1 Watt per element for six to seven minutes.

## 3. Results

**Figure 5:**
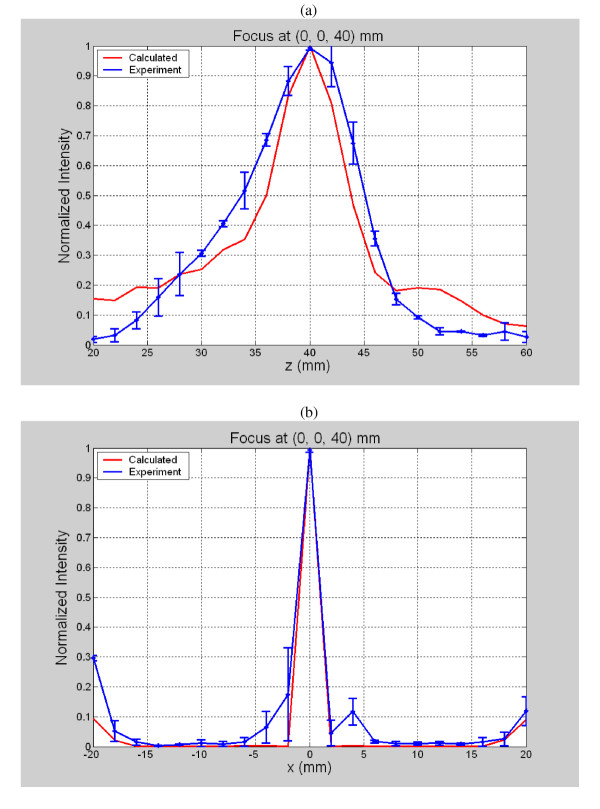
Comparison of calculated and experimental normalized intensities for a focus at 0, 0, 40 mm plotted along the (a) z axis and (b) x axis.

**Figure 6:**
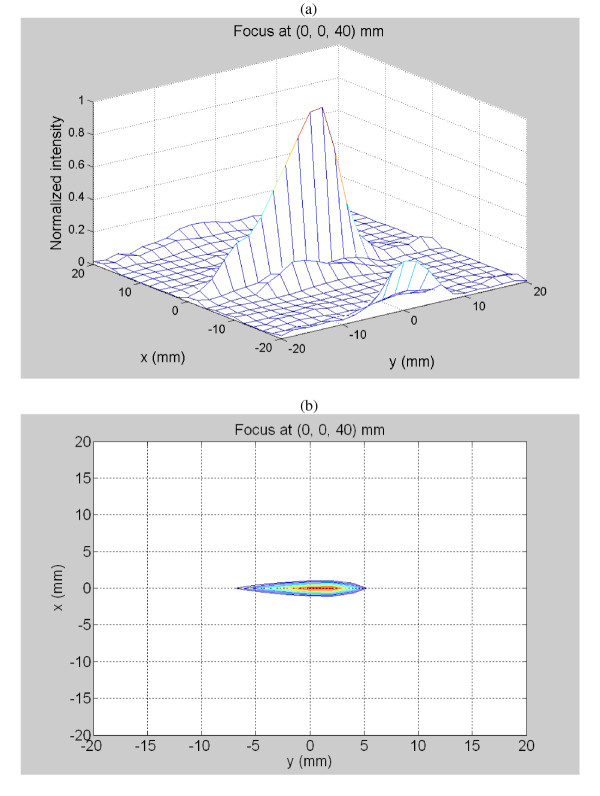
Exposimetry results of the normalized intensity for off-axis focusing with the focal point aimed at 0, 0, 40 mm plotted as a (a) mesh or (b) contour with levels indicated at 0, -1, -2, -3, -6 and -9 dB. These results indicate acceptable grating lobes of less than -9 dB.

To compare experimental and theoretical results, more than fifty exposimetry experiments were performed throughout the desired ablation volume to determine the focusing capability of the array. As an example of a typical exposimetry result at the location (x, y, z) = (0, 0, 40) mm, Figure 5(a) shows a comparison plot along the z-axis of the calculated and experimental normalized intensities. Figure 5(b) plots similar theoretical and experimental data but instead along the x-axis for the same focus (0, 0, 40 mm). As can be seen for both plots, the theoretical intensity data correlated well with the experimental results. To evaluate the feasibility of the array to steer the focus, a typical three dimensional normalized intensity result from a focal point directed at 0, 0, 40 mm. The results were plotted as a mesh (Figure 6(a)) and contour (Figure 6(b)) with contour levels at 0, -1, -2, -3, -6 and -9 dB of the normalized intensity with the grating lobe levels at about -9.0 dB or less.

**Figure 7:**
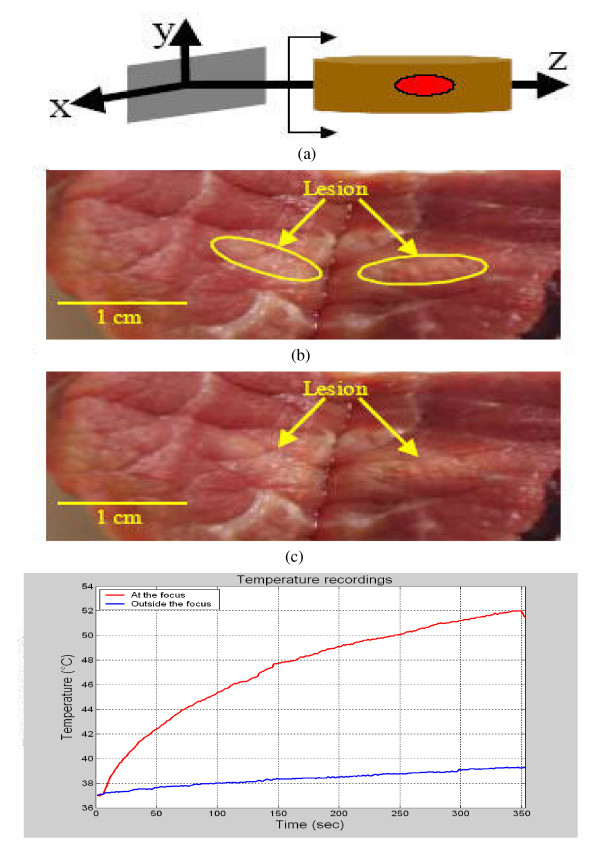
(a) A lesion, with dimensions of about 1 cm × 0.3 cm, generated by a six minute sonication time experiment (b) Temperature recordings at the locations of the focal point and grating lobe.

**Figure 8:**
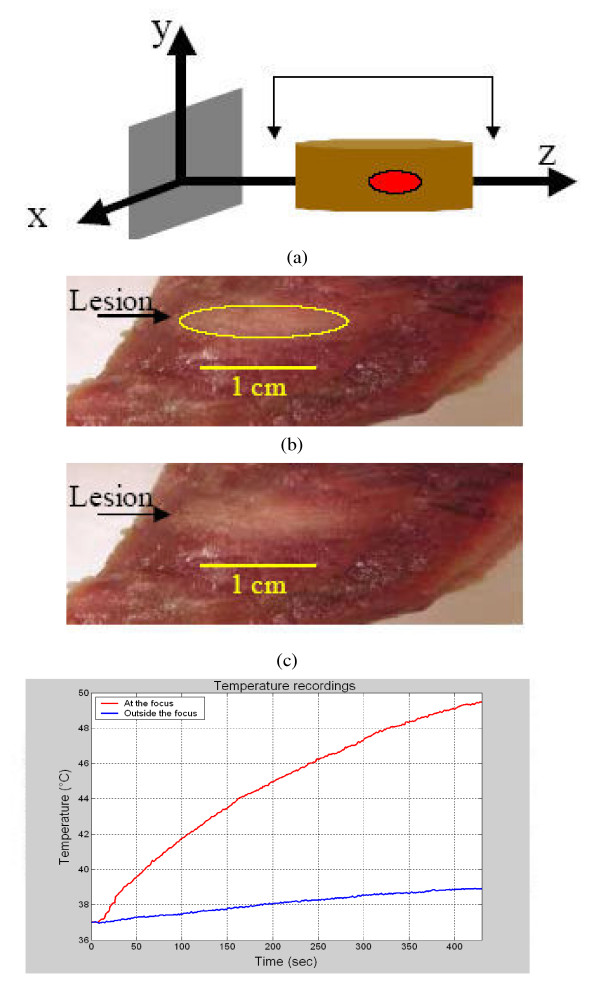
A marked (a) and unmarked (b) lesion, with dimensions of 1.3 cm × 0.38 cm, generated by a seven minute sonication time experiment, and the temperature recordings (c) at the locations of the focal point and grating lobe.

*Ex vivo *experiments were also performed to verify the ability of the array to generate lesions in bovine tissue. In one experiment, the array was turned on for about six minutes. After turning the array off, the bovine piece was cut at the position where the focal point was aimed. A lesion that has the dimensions of 1 cm × 0.3 cm was observed, as Figure 7(a) shows. An unmarked version of Figure 7(a) is shown in Figure 7(b) for better visualization of the lesion. During the experiment, thermocouples were used to monitor the temperature at two locations, the focal point and the grating lobe locations. The temperature recording, Figure 7(c), shows that the focal point temperature reached about 52°C, while the grating lobe temperature was kept below 40°C, as compared to the simulated values (using the BHTE) of 51.7°C and 40.2°C, respectively. In another experiment, the array was turned on for about seven minutes and then turned off. The observed lesion was approximately 1.3 cm × 0.38 cm in size, as shows in the marked picture of the lesion, Figure 8(a). The same picture, but without marking the lesion, is shown in Figure 8(b) for better visualization of the lesion. The temperature recordings for this experiment show that the temperature at the focal point position increased to reach about 49.5°C while the temperature at the grating lobe position was about 39°C at the end of the experiment, as shown in Figure 8(c). Although the sonication time for this experiment was seven minutes while it was six minutes for the experiment shown in Figure 8, the final temperature value at the focal point was less for the seven minute experiment as compared to the six minute one. This might be due to the uncertainty of the location of the thermocouples.

## 4. Discussion

Intracavitary ultrasound offers an attractive means of focused ultrasound treatment for BPH with significant advantages over other treatment methods due to the relatively short treatment time, its noninvasive nature and reduced complications. One compelling reason for using an intracavitary device with focused ultrasound is that the prostate is easily accessible via transrectal applicators, which allow for heating of the target volume in the prostate with minimal heating of normal tissue. Using phased arrays to electrically focus the ultrasound beam provides a controlled localized power deposition into tissue and reduces significantly the treatment time since the focus is electronically scanned instead of manually.

In designing this array, several issues were taken into account to address its application for BPH treatment. The dimensions of array were designed for an intracavitary rectal device. With appropriate housing, the array dimensions of 2.7 × 5.3 cm^2 ^are suitable. Another issue concerning this design was the grating lobe level, which was reduced significantly by tapering the array element lengths.

To treat the prostate, the array was aimed toward the intended target volume, and the elements were driven at a calculated amplitude and phase to generate a single focal point with electrical steering.

The array can ablate a volume that lies in its steering volumes. Assuming that the volume to be ablated is 1 × 1 × 2 cm^3 ^and that its center is 3 cm away from the array face, two techniques can be used as a treatment plan to ablate the whole volume; the first one is using a single focal point regime in which the target volume is divided into small volumes. The size of these small volumes is chosen based on the size of the lesion and the sonication time. Assuming that the lesion was a 4 mm long cigar shape with 2 mm diameter for a 10 second sonication, dividing the 1 × 1 × 2 cm^3 ^volume into 5 × 5 × 5 points indicates that 125 sonications are needed to ablate the target based on a single 10 second sonication that is electronically steered between the 125 positions. Starting at the center of the target volume, a single focal point is generated there and then electronically steered 125 times to cover the whole volume. To avoid uncontrolled heat buildup and pre-focal heating, the switching between the focal points is done in a way that any two focal points consecutive in time are far away from each other in distance. By doing that, enough time is given to the pre-focal positions to cool down. A second technique to ablate a large volume is by generating multi-focal points at the same time. Dividing the array into three areas, each responsible for generating a single focal point, will result in reducing the overall treatment time by a factor of three. This technique is time efficient, but the drawback behind it is that the driving electrical power per unit area should be increased.

If the maximum possible steering angle is 13.5° in the transverse direction, as the case for this array design, attempting to focus outside this volume will add a significant amount of energy to the grating lobes which will cause an unwanted heating. This puts a limitation for the array if the target volume extends beyond the 13.5° limit.

When coupled with MR temperature mapping, FUS provides an efficient way to treat BPH and at the same time gives a quick feedback about the temperature distribution inside the prostate [8]. Although ultrasound imaging for the prostate has shown to give good results [5,17], the array described here was designed to accompany an MRI.

Similar to prostate cancer treatment with focused ultrasound, benign fibroadenomas in the breast are currently treated clinically using multiple sonications from a single element transducer, which is mechanically scanned [18], in conjunction with MRI for guidance of thermotherapy of the procedure [19]. Although the treatment has been shown to be effective, the process includes an unnecessary delay due to the mechanical scanning protocol. When closely spaced locations are targeted with focused ultrasound, thermal buildup results from the accumulation of neighboring sonications and the nearfield heating. As a result, a lengthy delay between sonications (cooling time) is required to reduce thermal buildup. Investigators have shown that a cooling time of 50–60 seconds was necessary to reduce the heat from the near neighbor sonications [20] however this can add considerable time to the procedure and initiate inaccuracies to the MR thermometry through patient motion. With phased arrays a focal pattern can be arranged such that there is enough time for the heat to dissipate by sonicating non-neighboring regions within the tumor [21]. A treatment planning routine can be plotted over the entire tumor region such that the volume is ablated through distant and non-adjacent ablations to avoid thermal buildup yet destroy the volume in the least amount of time. This research demonstrates the feasibility of electrically steered arrays that can be used to ablate tissue for the intended treatment of benign prostatic hyperplasia.

## 5. Conclusion

A 1.75-D ultrasound phased array, that can focus and steer in a 3-D representation of the prostate without the need to physically move the array, had been successfully built and tested for the treatment of prostate cancer and BPH. Previous focused ultrasound array designs were problematic since they required complex methods to move the focus, as for the case of annular arrays, or had linear (1-D) designs that were only capable of focusing along one axis. These drawbacks were the motivation to design a new array that can be used in FUS and at the same time be systematically controlled to reposition the focus throughout a specific volume with an acceptable level of grating lobes. Further improvement over this array design seems to be feasible due to recent developments in building focused transducers using piezocomposite technology [22]. A three-layer PZT-8 material may also be used to increase the capacitance and thus make it easier to electrically match the small elements.

## 6. Authors' contributions

The first author (Saleh) contributed 60% of the work done for this paper. He performed the computer simulations required for the study, participated in performing the experiments that were performed to verify the study, and participated in the drafting and revising of the article. The other Author (Smith) contributed 40% of work done. She came up with idea, participated in some of the experiments performed and participated in the drafting and revising of the article. So both authors have made substantial contributions, have been involved in writing the article and have given final approval for the final submitted version.
